# USP7‐Mediated ICAM1 Facilitates Lipopolysaccharide‐Induced Human Pulmonary Microvascular Endothelial Cell Injury to Accelerate Pediatric Acute Respiratory Distress Syndrome

**DOI:** 10.1111/crj.70079

**Published:** 2025-05-06

**Authors:** Jing Li, Jing Wu, Lili Zhao, Lian Liu

**Affiliations:** ^1^ Department of Neonatology Chongqing Bishan District Maternal and Child Health Hospital Chongqing China; ^2^ Department of Pediatrics University‐Town Hospital of Chongqing Medical University Chongqing China

**Keywords:** ICAM1, pediatric acute respiratory distress syndrome, USP7

## Abstract

**Background:**

Intercellular cell adhesion molecule 1 (ICAM1) has been confirmed to be abnormally expressed in acute respiratory distress syndrome (ARDS) patients. However, its role and mechanism in pediatric ARDS process need further revealed.

**Methods:**

Serum samples were selected from pediatric ARDS patients and age‐matched healthy individuals. Lipopolysaccharide (LPS)‐induced human pulmonary microvascular endothelial cells (HPMECs) were used to mimic ARDS cell models. Cell proliferation and apoptosis were tested by cell counting kit 8 assay, EdU assay, and flow cytometry. Oxidative stress and inflammation were assessed by corresponding kits. M1 macrophage polarization was evaluated via measuring CD86 positive cell rate. The expression levels of ICAM1, ubiquitin‐specific peptidase 7 (USP7), and NF‐κB pathway‐related markers were detected by quantitative real‐time PCR and western blot. The interaction between USP7 and ICAM1 was analyzed by Co‐IP assay.

**Results:**

LPS induced apoptosis, inflammation, oxidative stress, and M1 macrophage polarization, while suppressed proliferation in HPMECs. ICAM1 was upregulated in pediatric ARDS patients, and its knockdown alleviated HPMEC injury induced by LPS. USP7 positively regulated ICAM1 protein expression through deubiquitination. USP7 overexpression aggravated LPS‐induced HPMEC apoptosis, inflammation, oxidative stress, and M1 macrophage polarization. Besides, ICAM1 upregulation could eliminate the inhibitory effect of USP7 knockdown on LPS‐induced HPMEC injury. In addition, USP7 activated NF‐κB pathway by promoting ICAM1 expression.

**Conclusion:**

USP7‐mediated ICAM1 upregulation could promote LPS‐induced HPMEC injury by activating NF‐κB pathway, which provided a new idea for the treatment of pediatric ARDS.

## Graphical abstract

USP7‐mediated ICAM1 induced apoptosis, inflammation, oxidative stress, and M1 macrophage polarization, while suppressed proliferation in LPS‐induced HPMECs via activating NF‐κB pathway, thus accelerating the progression of pediatric acute respiratory distress syndrome.

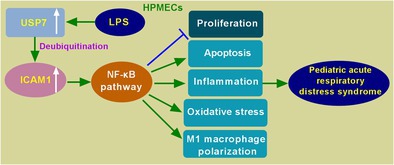

## 1 Introduction

Pediatric acute respiratory distress syndrome (ARDS) is caused by a variety of primary diseases, such as infantile pneumonia, sepsis, post‐cardiopulmonary resuscitation, septic shock, aspiration, and drowning [1, 2]. ARDS is mainly manifested as acute respiratory distress and refractory hypoxemia in children, which seriously affects the lives of children [3, 4]. The basic pathological changes of the lung in ARDS are acute diffuse injury of pulmonary vascular endothelium and lung epithelium [5, 6]. At present, human pulmonary microvascular endothelial cells (HPMECs) induced by lipopolysaccharide (LPS) are considered as one of the in vitro models to carry out ARDS‐related research [7, 8]. Therefore, understanding the molecular mechanism of LPS‐induced HPMEC injury may provide new ideas for the treatment of pediatric ARDS.

Intercellular cell adhesion molecule 1 (ICAM1), a member of the immunoglobulin superfamily, is a vital adhesion molecule that mediates adhesion reactions, which is usually expressed on endothelial cells and immune cells [9, 10]. ICAM1 plays an important role in regulating endothelial function, promoting adhesion of inflammatory sites and controlling tumor metastasis [11, 12, 13]. Many studies have shown the protective effect of ICAM1 on endothelial cell injury. Studies had revealed that silencing of ICAM1 reduced hypoxia/reoxygenation‐induced cardiac microvascular endothelial cell injury [14], and its overexpression promoted ox‐LDL‐stimulated inflammation and apoptosis in human umbilical vein endothelial cells [12]. In this study, we found that ICAM1 was overexpressed in LPS‐stimulated HPMECs by GEO database (GSE5883) analysis. Importantly, Yao et al. suggested that ICAM1 was upregulated in ARDS patients, and its downregulation suppressed inflammation and apoptosis in LPS‐induced HPMECs [15]. Therefore, ICAM1 is likely to be potential target of ARDS treatment, and its role and molecular mechanism in ARDS process deserve further reveal.

Ubiquitination is an important post‐translational modification that is widely involved in the regulation of various biological processes, such as cell metabolism, cell cycle, and tumorigenesis [16, 17]. Ubiquitin‐specific peptidase 7 (USP7) is a member of the deubiquitinating enzyme family, which mainly regulates cellular functions, including cell damage repair and inflammatory response [18, 19]. Previous studies had revealed that USP7 inhibitor P22077 could suppress inflammation and alleviate lung injury in LSP‐induced mice models by inhibiting NF‐κB and MAPKs pathways [20]. Therefore, USP7 may be a potentially important regulator of lung injury‐related diseases, including ARDS. Here, we discovered that USP7 had increased expression in pediatric ARDS patients. Through the prediction of ubibrowser database, we found that USP7 could regulate ICAM1 by deubiquitination, and further studies confirmed that USP7 could affect the protein expression of ICAM1. However, whether USP7 regulates ARDS process by mediating the deubiquitination of ICAM1 is unknown.

In this study, we aimed to reveal the underlying molecular mechanism by which ICAM1 regulated pediatric ARDS progression using LPS‐induced HPMEC models. Based on the above, we proposed and confirmed the hypothesis that USP7‐mediated ICAM1 by deubiquitination, which in turn promoted LPS‐induced HPMEC injury.

## 2 Materials and Methods

### 2.1 Samples

Pediatric ARDS patients (*n* = 31) who were hospitalized at Chongqing Bishan District Maternal and Child Health Hospital from July 2018 and January 2020 were selected. Peripheral blood (2 mL) was collected from patients on the day of diagnosis of ARDS, which were then centrifuged for obtaining serum samples. Meanwhile, 27 age‐matched healthy individuals (undergoing physical examination) were recruited and their serum samples were collected as normal control. The basic clinical data of participants are listed in Table 1. The families of all participants signed written informed consent, and this study was obtained by the Ethics Committee of Chongqing Bishan District Maternal and Child Health Hospital.

**TABLE 1:** The basic clinical data of participants.

Parameters	Healthy individuals (*n* = 27)	Pediatric ARDS (*n* = 31)	*p* value
Gender (male/female)	15/12	17/14	>0.9999
Age (years)	3.82 ± 1.53	3.74 ± 2.04	0.8681
IL‐6 (pg/mL)	10.54 ± 1.73	23.47 ± 2.58	<0.0001
TNF‐α (pg/mL)	18.63 ± 3.64	46.37 ± 4.85	<0.0001
PICU length of stay (mean ± SD)	NA	14.23 ± 5.47	
Days of oxygen therapy (mean ± SD)	NA	15.87 ± 8.41	

### 2.2 Cell Treatment and Transfection

HPMECs (ScienCell, San Diego, California, United States) were cultured in endothelial culture medium at 37 °C with 5% CO_2_, which were treated with different concentrations (200, 400, and 800 ng/mL) of LPS (Solarbio, Beijing, China) for 24 h to explore the effect of LPS on HPMEC injury. HPMECs were transfected with the siRNAs and pcDNA overexpression vector of ICAM1 and USP7 (si‐ICAM1 and si‐USP7) in six‐well plates (reached 50% confluences) using Lipofectamine 3000 (Invitrogen, Carlsbad, California, United States).

Monocytes (THP‐1; Biovector, Beijing, China) were grown in RPMI‐1640 plus 0.05 mM β‐mercaptoethanol, 10% FBS and 1% P/S (Gibco, Grand Island, NY, USA). THP‐1 cells were treated with PMA (100 ng/mL, Sangon, Shanghai, China) for 24 h to induce M0 macrophages. In co‐culture system, THP‐1 cells treated with PMA were seeded in the upper of transwell chambers (Corning Inc., Corning, New York, USA), while HPMECs were seeded into the lower chambers.

### 2.3 Cell Counting kit 8 (CCK8) Assay

According to the instructions of CCK8 Kit (Beyotime, Shanghai, China), HPMECs in 96‐well plates were incubated with CCK8 reagent for 2 h. Absorbance measurements were performed using a microplate reader at 450 nm to assess cell viability.

### 2.4 EdU Assay

HPMECs in 96‐well plates were incubated with EdU solution, fixed by paraformaldehyde, decolored by glycine, treated with TritonX‐100, and stained with Apollo solution using EdU Kit (RiboBio, Guangzhou, China). After incubated with DAPI for nuclear staining, EdU‐positive cells were visualized under a microscope and counted by ImageJ software.

### 2.5 Flow Cytometry

For measuring cell apoptosis rate, HPMECs were collected and centrifuged. After resuspended in binding buffer, cells were double‐stained with Annexin V‐FITC and PI (Solarbio), followed by analyzed cell apoptosis rate using flow cytometer.

For detecting CD86‐positive (CD86^+^) cell rate, M0 macrophages in co‐cultured system were collected and incubated with FITC‐labeled anti‐CD86 (ab77276, Abcam, Cambridge, California, United States), followed by analyzed CD86^+^ cell rate using flow cytometer.

### 2.6 Detection of Oxidative Stress

ROS levels and SOD activity in HPMECs were detected by ROS Assay Kit and SOD Assay Kit (all from Jiancheng Biotechnology Co., Ltd., Nanjing, China) according to kit instructions.

### 2.7 ELISA

The culture medium of HPMECs was collected and centrifuged for obtaining cell supernatant. The levels of inflammation factors (IL‐6, IL‐1β, and TNF‐α) were detected by corresponding ELISA Kits (Multisciences, Hangzhou, China) basing on kit instructions.

### 2.8 Quantitative Real‐Time PCR (qRT‐PCR)

Total RNA was extracted using TRIzol reagent (Invitrogen). After measuring concentrations, cDNA was obtained using PrimeScript RT Reagent Kit (TaKaRa, Dalian, China). PCR was performed using SYBR Green (TaKaRa) with cDNA and specific primers (Table 2). Relative expression was analyzed using 2^−ΔΔCt^ method.

**TABLE 2:** Primer sequences used for qRT‐PCR.

Name		Primers for PCR (5′‐3′)
USP7	Forward	CCGAGGACATGGAGATGGAAG
	Reverse	TCACTCAGTCTGCTGAAGCG
ICAM1	Forward	TCTTCCTCGGCCTTCCCATA
	Reverse	AGGTACCATGGCCCCAAATG
GAPDH	Forward	GGAGCGAGATCCCTCCAAAAT
	Reverse	GGCTGTTGTCATACTTCTCATGG

### 2.9 Western Blot (WB)

Total protein was extracted by RIPA buffer and qualified by BCA Kit (Beyotime). Proteins were loaded on 10% SDS‐PAGE gel and transferred to PVDF membranes. After blockage, membranes were incubated with appropriate primary and secondary antibodies. Immunoreactive bands were visualized by ECL reagent and analyzed with ImageJ software. Antibodies (all from Abcam) included anti‐ICAM1 (1:2000, ab109361), anti‐USP7 (1:2000, ab4080), anti‐p‐P65 (1:1000, ab76302), anti‐P65 (1:1000, ab32536), anti‐p‐IκBα (1:1000, ab133462), anti‐IκBα (1:10000, ab32518), anti‐GAPDH (1:2500, ab9485), and goat anti‐rabbit (1:50000, ab205718).

### 2.10 Co‐IP Assay

In accordance with the instructions of Co‐Immunoprecipitation Kit (Pierce, Rockford, Illinois, United States), HPMEC lysates were incubated with anti‐IgG, anti‐USP7, anti‐ICAM1, and protein A/G agarose beads. The precipitated proteins were obtained for WB. Besides, HPMECs were transfected with Flag‐ICAM1, si‐NC or si‐USP7, and then the cell lysates were incubated with anti‐USP7, HA‐Ub, and protein A/G agarose beads. Following, the precipitated protein was collected for WB.

### 2.11 Statistical Analysis

Data are represented as mean ± SD. Statistical analysis was conducted using GraphPad Prism 7.0. Differences were assessed by Student's *t* test or ANOVA. *p* < 0.05 was statistically significant.

## 3 Results

### 3.1 LPS Induced HPMEC Apoptosis, Oxidative Stress, Inflammation, and M1 Macrophage Polarization

**FIGURE 1:**
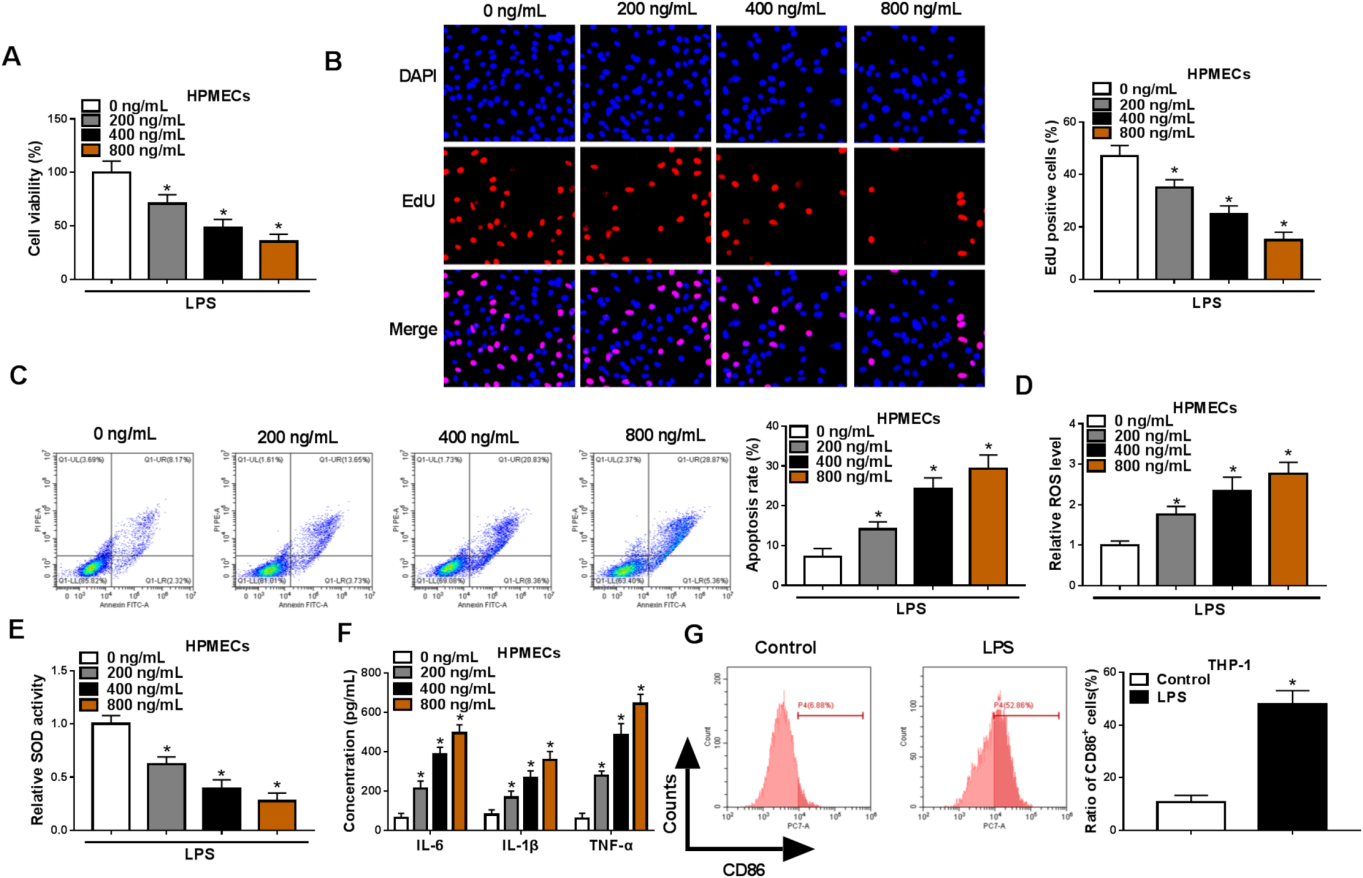
Effect of LPS on HPMEC functions. (A–F) HPMECs were treated with different concentrations of LPS. CCK8 assay (A), EdU assay (B), and flow cytometry (C) were used to measure cell proliferation and apoptosis. (D,E) ROS levels and SOD activity were detected to assess oxidative stress. (F) Inflammation factor levels were examined by ELISA. (G) Flow cytometry was performed to evaluate CD86^+^ cell rate in M0 macrophages (PMA‐induced THP‐1 cells) co‐cultured with LPS‐induced HPMECs. (A–E) One‐way ANOVA; (F) two‐way ANOVA; (G) Student's *t* test. *p < 0.05.

Firstly, we measured HPMEC functions treated with different concentrations of LPS. The viability, EdU‐positive cell rate, and SOD activity of HPMECs were gradually decreased with the increasing of LPS concentration, while the apoptosis rate and ROS levels were significantly enhanced by LPS in a concentration‐dependent manner (Figure 1A–E). Also, LPS treatment remarkably promoted the levels of IL‐6, IL‐1β, and TNF‐α in a dose‐dependent manner (Figure 1F). M1 macrophage polarization is associated with the secretion of inflammatory factors [21, 22]. Here, we examined the effect of LPS on M1 macrophage polarization, and the results showed that the cell rate of M1 macrophage marker CD86^+^ was markedly increased in M0 macrophages co‐cultured with LPS‐induced HPMECs (Figure 1G). These results indicated that LPS‐treated HPMECs induced M1 macrophage polarization.

### 3.2 ICAM1 Was Overexpressed in Pediatric ARDS Patients and LPS‐Induced HPMECs

**FIGURE 2:**
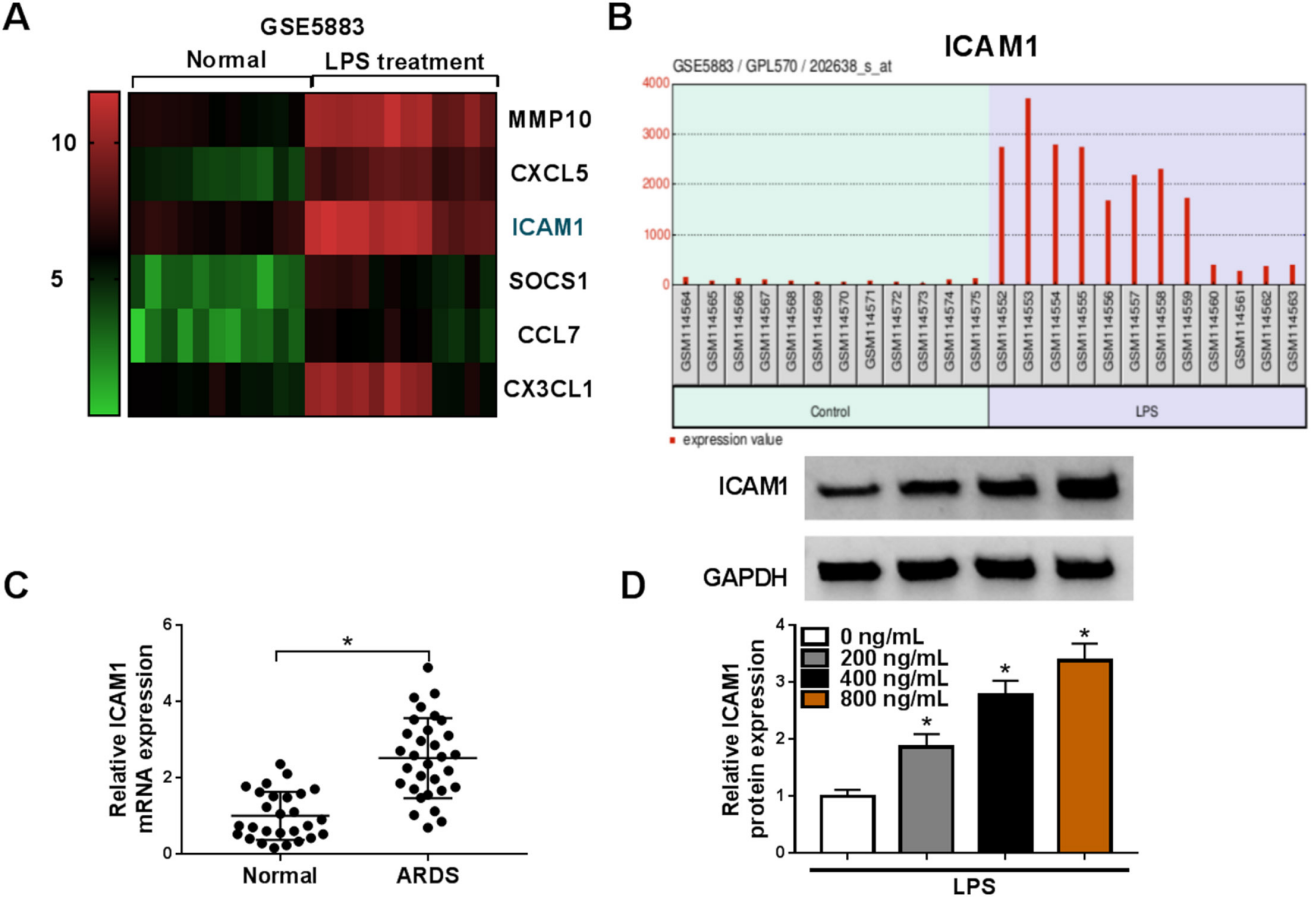
ICAM1 expression in pediatric ARDS patients and LPS‐induced HPMECs. (A) Heatmap showed the differentially expressed genes in HPMECs treated with or without LPS (*n* = 12) in GSE5883. (B) ICAM1 expression in HPMECs treated with or without LPS (*n* = 12) in GSE5883 was shown. (C) ICAM1 mRNA expression was detected by qRT‐PCR in pediatric ARDS patients (*n* = 31) and healthy individuals (*n* = 27). (D) ICAM1 protein expression in HPMECs treated with different concentrations of LPS was analyzed by WB. (C) Student's *t* test; (D) one‐way ANOVA. *p < 0.05.

Through analyzing GEO database (accession: GSE5883, criteria: *p* < 0.001 and|log2FC| > 3.0), we confirmed that ICAM1 was upregulated in LPS‐induced HPMECs (Figure 2A,B). To further confirm this, we detected ICAM1 expression in pediatric ARDS patients and LPS‐induced HPMECs. Compared to healthy individuals, ICAM1 was significantly higher in the serum of pediatric ARDS patients (Figure 2C). Moreover, WB analysis showed that the protein expression of ICAM1 was increased in HPMECs with the increasing concentration of LPS (Figure 2D).

### 3.3 ICAM1 Knockdown Alleviated LPS‐Induced HPMEC Injury

**FIGURE 3:**
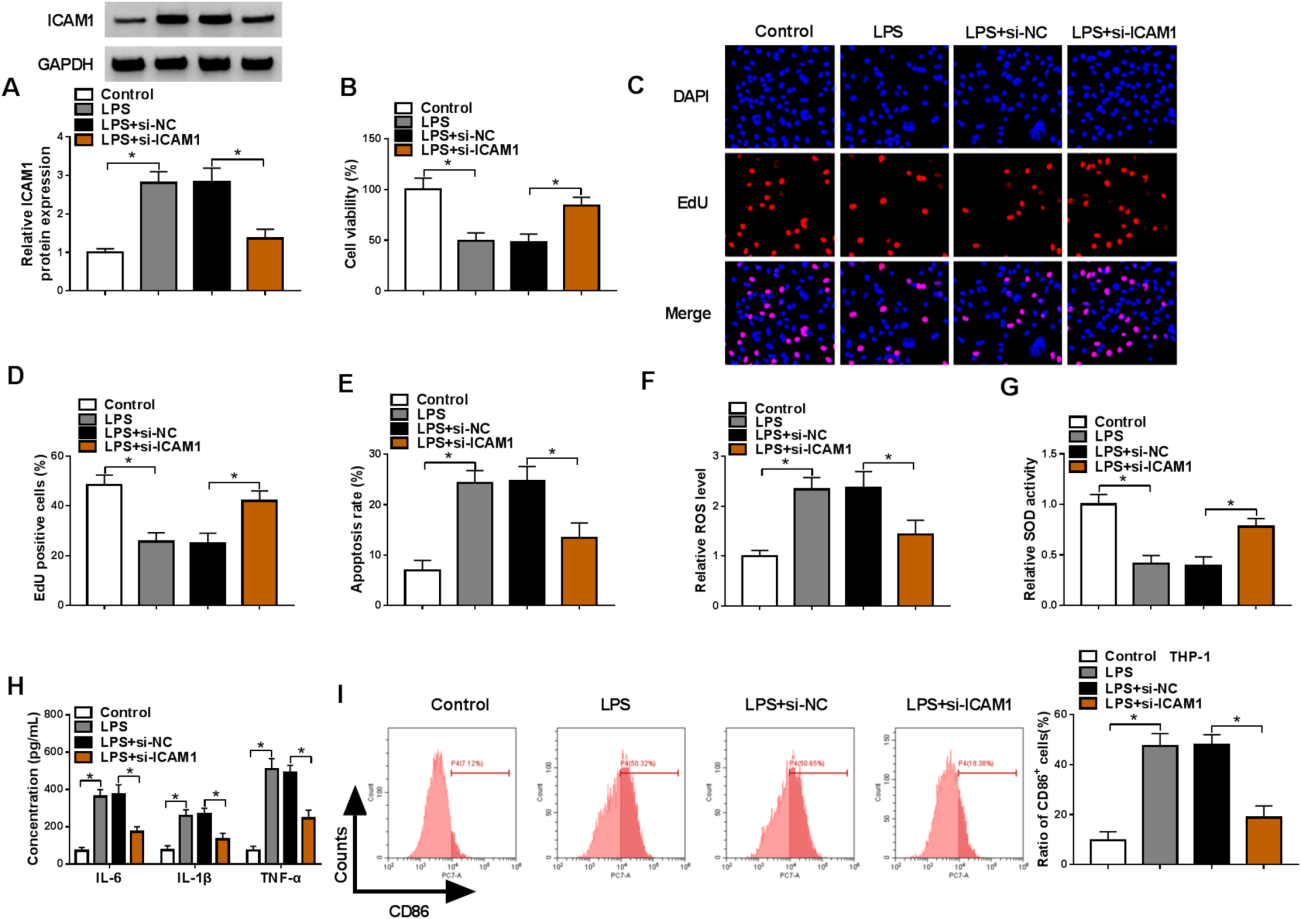
Effect of si‐ICAM1 on LPS‐induced HPMEC injury. (A–H) HPMECs were transfected with si‐NC/si‐ICAM1 and then treated with 400 ng/mL LPS. (A) ICAM1 protein expression was detected by WB. Cell proliferation and apoptosis were examined using CCK8 assay (B), EdU assay (C,D), and flow cytometry (E). (F,G) Oxidative stress was assessed by detecting ROS levels and SOD activity. (H) ELISA was used to test inflammation factor levels. (I) CD86^+^ cell rate in co‐cultured M0 macrophages was analyzed by flow cytometry. (A–G,I) One‐way ANOVA; (H) two‐way ANOVA. *p < 0.05.

For revealing ICAM1 roles in ARDS progression, we silenced ICAM1 using si‐ICAM1 in LPS‐induced HPMECs. The decreased ICAM1 protein expression in LPS‐induced HPMECs confirmed the successful transfection of si‐ICAM1 (Figure 3A). ICAM1 knockdown enhanced viability, EdU‐positive cell rate, and SOD activity, while it reduced apoptosis rate and ROS levels in LPS‐induced HPMECs (Figure 3B–G). Besides, the levels of IL‐6, IL‐1β, and TNF‐α in LPS‐induced HPMECs could be decreased by ICAM1 knockdown (Figure 3H). In addition, downregulation of ICAM1 also decreased CD86^+^ cell rate of M0 macrophages co‐cultured with LPS‐induced HPMECs (Figure 3I).

### 3.4 Deubiquitinating Enzyme USP7 Affected ICAM1 Protein Expression

**FIGURE 4:**
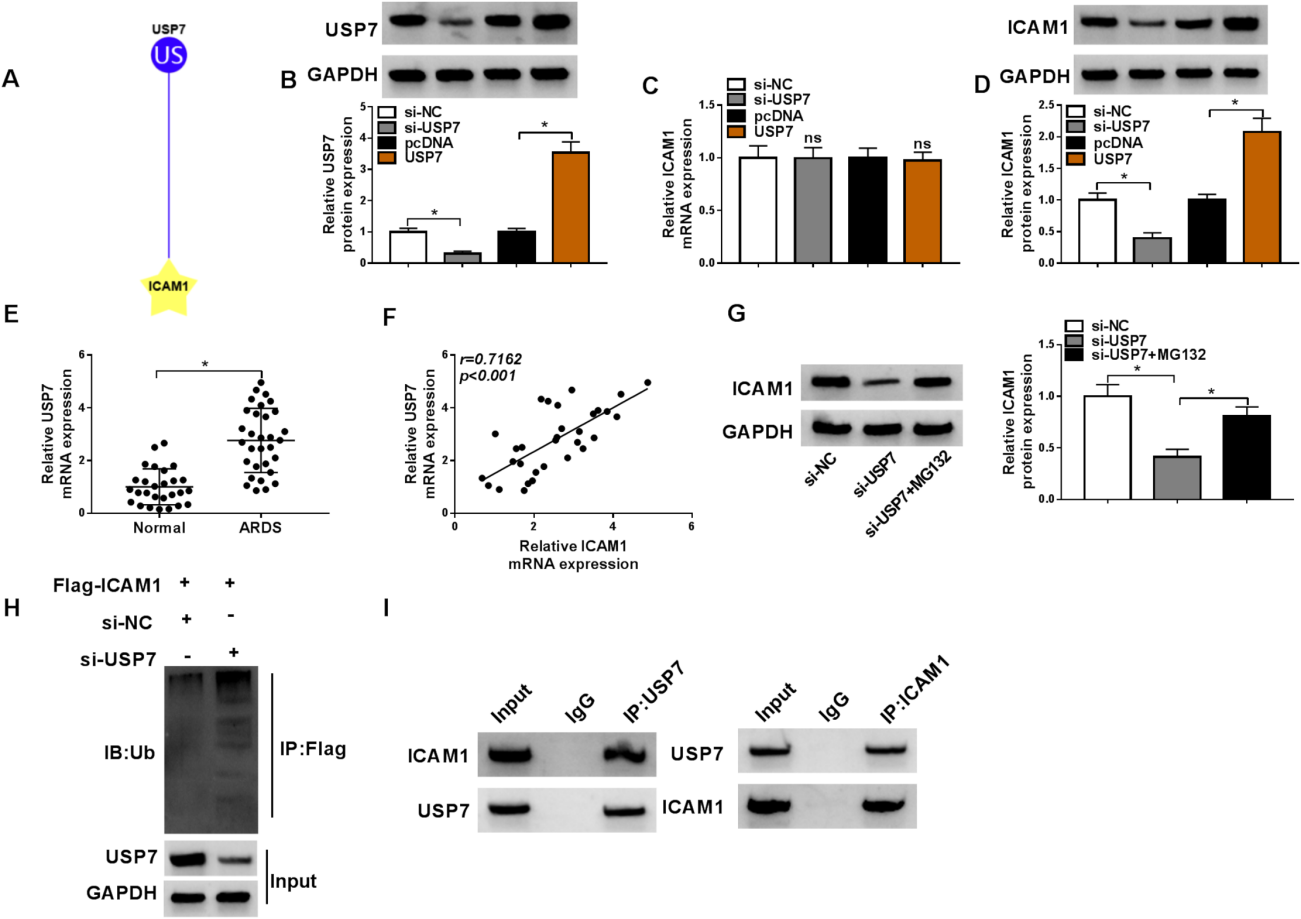
Effect of USP7 on ICAM1 protein expression. (A) The ubibrowser database predicted that USP7 deubiquitinated ICAM1. (B) WB confirmed the transfection efficiencies of si‐USP7 and USP7 overexpression vector. (C,D) ICAM1 mRNA and protein levels were tested by qRT‐PCR and WB in HPMECs transfected with si‐USP7/si‐NC/pcDNA/USP7. (E) USP7 mRNA expression was detected by qRT‐PCR in pediatric ARDS patients (*n* = 31) and healthy individuals (*n* = 27). (F) The correlation between USP7 and ICAM1 expression was confirmed by Pearson correlation analysis. (G) ICAM1 protein expression in HPMECs transfected with si‐NC/si‐USP7 and treated with MG132 (10 μmol/L) was analyzed by WB. (H,I) Co‐IP assay confirmed the interaction between USP7 and ICAM1. (B–D,G) One‐way ANOVA; (E) Student's *t* test. *p < 0.05.

Furthermore, we found that USP7 could deubiquitinate ICAM1 using ubibrowser database prediction (Figure 4A). In further analysis, USP7 protein expression was decreased by si‐USP7 and increased by USP7 overexpression vector (Figure 4B). The detection of ICAM1 expression showed that USP7 knockdown and overexpression did not affect the mRNA level of ICAM1, but USP7 knockdown could inhibit ICAM1 protein level, and its overexpression had the opposite effect (Figure 4C,D). USP7 had elevated mRNA expression in pediatric ARDS patients, and its expression was positively correlated with ICAM1 expression (Figure 4E,F). Proteasome inhibitor MG132 reversed the decreasing effect of USP7 knockdown on ICAM1 protein expression, confirming that USP7 could stabilize ICAM1 protein (Figure 4G). Importantly, Co‐IP assay suggested that USP7 knockdown could increase the ubiquitination of ICAM1 protein (Figure 4H), and USP7 protein could bind to ICAM1 protein each other (Figure 4I).

### 3.5 Overexpression of USP7 Promoted LPS‐Induced HPMEC Injury

**FIGURE 5:**
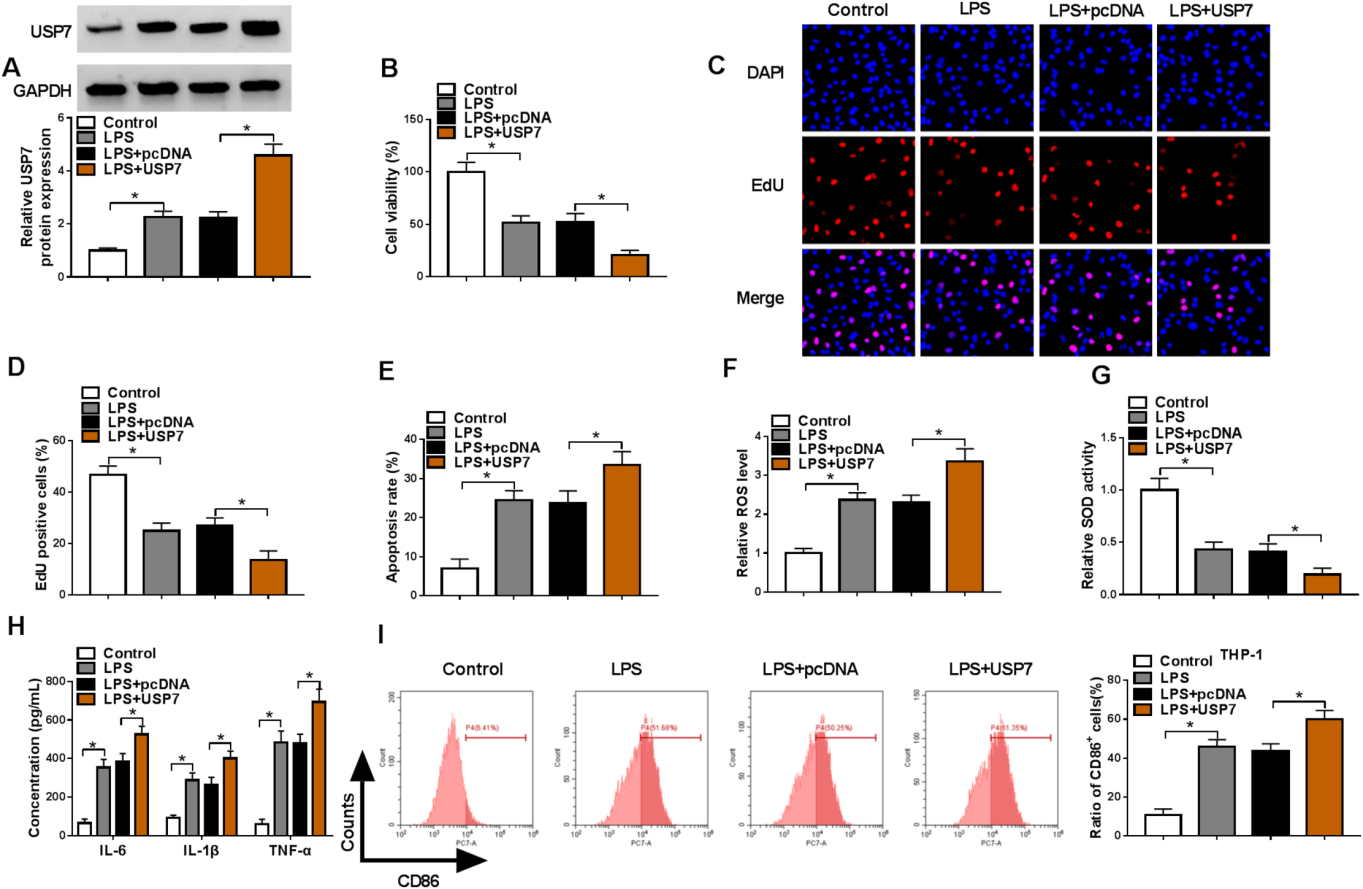
Effect of USP7 promoted LPS‐induced HPMEC injury. (A–H) HPMECs were transfected with pcDNA/USP7 overexpression vector and then treated with 400 ng/mL LPS. (A) USP7 protein expression was examined by WB. CCK8 assay (B), EdU assay (C,D), and flow cytometry (E) were performed to test cell proliferation and apoptosis. (F,G) ROS levels and SOD activity were measured to detect oxidative stress. (H) Inflammation factor levels were tested by ELISA. (I) Flow cytometry was used to examine CD86^+^ cell rate in co‐cultured M0 macrophages. (A–G,I) One‐way ANOVA; (H) two‐way ANOVA. *p < 0.05.

To further explore USP7 roles in ARDS progression, we performed functional experiments. After transfection of USP7 overexpression vector, USP7 protein expression was markedly enhanced in LPS‐induced HPMECs (Figure 5A). USP7 overexpression further aggravated the effect of LPS on HPMEC viability, EdU positive cell rate, apoptosis rate, ROS levels and SOD activity (Figure 5B–G). Moreover, USP7 overexpression promoted the levels of IL‐6, IL‐1β, and TNF‐α in LPS‐induced HPMECs, as well as CD86^+^ cell rate of M0 macrophages (Figure 5H,I).

### 3.6 ICAM1 Overexpression Reversed the Effect of si‐USP7 on LPS‐Induced HPMEC Injury

**FIGURE 6:**
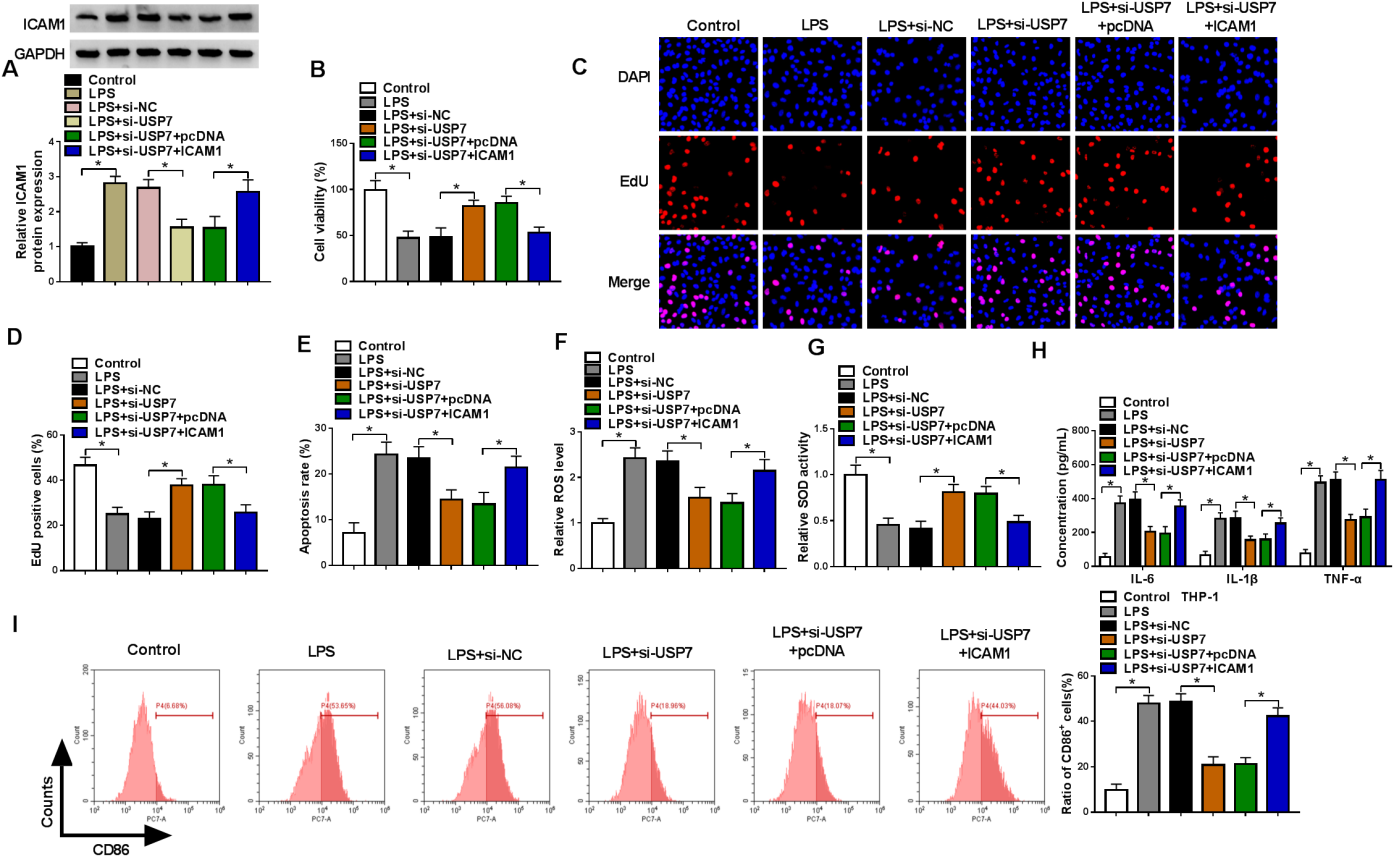
Effect of si‐USP7 and ICAM1 on LPS‐induced HPMEC injury. (A–H) HPMECs were transfected with si‐NC/si‐USP7/pcDNA/ICAM1 and then treated with 400 ng/mL LPS. (A) ICAM1 protein expression was measured using WB. Cell proliferation and apoptosis were detected by CCK8 assay (B), EdU assay (C,D), and flow cytometry (E). (F,G) Oxidative stress was evaluated via measuring ROS levels and SOD activity. (H) ELISA was performed to detect inflammation factor levels. (I) CD86^+^ cell rate in co‐cultured M0 macrophages was measured using flow cytometry. (A–G,I) One‐way ANOVA; (H) two‐way ANOVA. *p < 0.05.

Following, we explored whether USP7 interacted with ICAM1 to regulate HPMEC injury. The transfection of ICAM1 overexpression vector significantly increased ICAM1 expression suppressed by si‐USP7 in LSP‐induced HPMECs (Figure 6A). USP7 knockdown promoted viability and EdU‐positive cell rate in LSP‐induced HPMECs, and these effects were reversed by ICAM1 overexpression (Figure 6B–D). Besides, downregulation of USP7 inhibited apoptosis rate, ROS levels and promoted SOD activity in LPS‐induced HPMECs, whereas ICAM1 overexpression also abolished these effects (Figure 6E–G). Similarly, ICAM1 overexpression eliminated the reducing effect of USP7 silencing on the levels of inflammation factors and CD86^+^ cell rate (Figure 6H,I).

### 3.7 USP7 Regulated ICAM1 to Mediate NF‐κB Pathway

**FIGURE 7:**
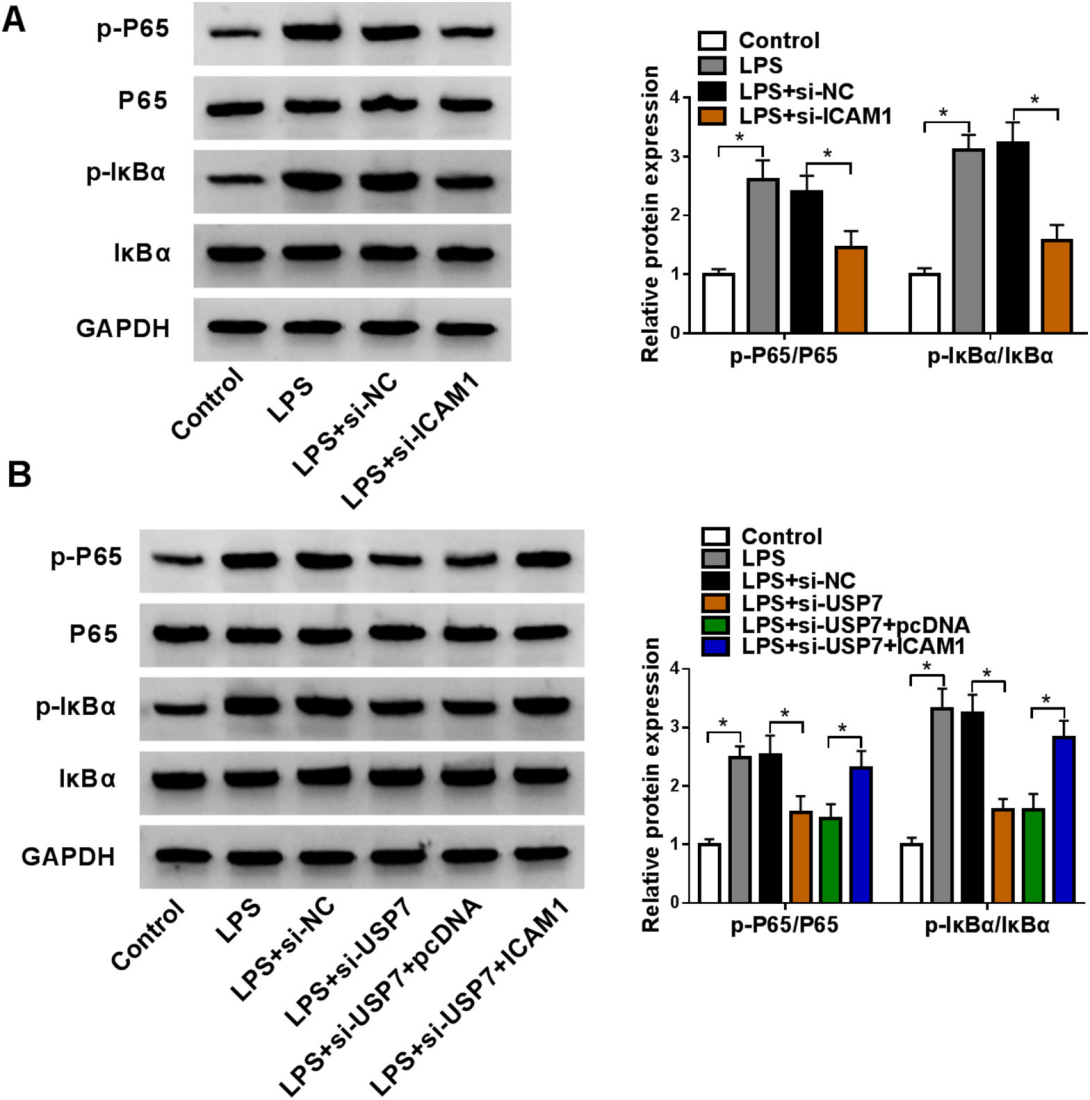
Effect of USP7/ICAM1 axis on the activity of NF‐κB pathway. (A) The protein levels of p‐P65/P65 and p‐IκBα/IκBα in HPMECs transfected with si‐NC/si‐ICAM1 and treated with 400 ng/mL LPS were examined by WB. (B) The protein levels of p‐P65/P65 and p‐IκBα/IκBα were analyzed by WB in HPMECs transfected with si‐NC/si‐USP7/pcDNA/ICAM1 and treated with 400 ng/mL. (A,B) Two‐way ANOVA. *p < 0.05.

NF‐κB pathway is confirmed to be involved in ARDS progression [23, 24]. In this, we measured the activity of NF‐κB pathway. LPS induction markedly increased the protein levels of p‐P65/P65 and p‐IκBα/IκBα in HPMECs, while ICAM1 knockdown reversed this effect (Figure 7A). USP7 knockdown also decreased the protein levels of p‐P65/P65 and p‐IκBα/IκBα in LPS‐induced HPMECs, and ICAM1 overexpression could eliminate this effect (Figure 7B).

## 4 Discussion

Pediatric ARDS is one of the most common critical diseases of the respiratory system in children, with a high mortality rate [25]. More and more studies have shown that endothelial cell injury is an important marker of ARDS, and its degree is closely related to patients' prognosis [5, 26]. Therefore, clarifying the molecular mechanism of endothelial cell injury may provide new way for ARDS treatment. In this, we constructed ARDS cell injury models in vitro using LPS‐induced HPMECs. We reveal that USP7‐mediated ICAM1 promotes LPS‐induced HPMEC injury via the activating of NF‐κB pathway, which points to a novel mechanism for regulating ARDS progression.

Cell adhesion molecules are a class of membrane protein molecules that mediate the interaction between cells and extracellular matrix, and they are crucial in maintaining the normal structure and function of body tissues [27, 28]. ICAM1 is the most studied adhesion molecule, which plays a key role in human disease processes. ICAM1 overexpression had been confirmed to accelerate the metastasis of gallbladder cancer cells [29]. Besides, Liu et al. showed that ICAM1 was overexpressed in myocarditis patients, and its silencing could restrain LPS‐induced cardiomyocyte inflammation and apoptosis by inactivating NF‐κB pathway [30]. In addition, targeted inhibition of ICAM1 might be an effective treatment for hypertension, because ICAM1 knockdown could inhibit Ang II‐induced arterial hypertension and vascular hypertrophy [31]. Previous studies had shown that vaspin could repress the inflammatory response of HPMECs and endothelial cells to proinflammatory cytokines by reducing ICAM1 level and NF‐κB pathway activity [32, 33]. These data confirmed the pro‐inflammation and pro‐NF‐κB pathway roles of ICAM1. Consistent with the results of Yao et al. [15], our data verified the high ICAM1 expression in pediatric ARDS patients and confirmed the pro‐inflammation and pro‐apoptosis effects of ICAM1 on LPS‐induced HPMECs. Not only that, our study also suggested that ICAM1 knockdown promoted cell proliferation and inhibited oxidative stress in LPS‐induced HPMECs, and suppressed M1 macrophage polarization via inactivating NF‐κB pathway.

As a deubiquitinating enzyme, USP7 roles in human disease have been widely revealed. USP7 promoted the deubiquitination of EZH2, thereby accelerating prostate cancer cell metastasis [34]. Moreover, USP7 might contribute to osteoporosis, which enhanced osteoclast differentiation by deubiquitinating HMGB1 [35]. In this, we confirmed that USP7 could promote ICAM1 protein expression by deubiquitination. Previous study confirmed that USP7 aggravated hypoxia‐induced cardiomyocyte apoptosis and inflammation in myocardial infarction [36], and accelerated pyroptosis, oxidative stress, and inflammation of H_2_O_2_‐induced chondrocytes in osteoarthritis [37]. Although USP7 inhibitor P22077 has been proposed for the treatment of inflammatory diseases and lung injury [20], USP7 roles in ARDS progression are still unclear. Here, we detected the high USP7 expression in pediatric ARDS patients, and suggested that USP7 overexpression enhanced LPS‐induced HPMEC apoptosis, oxidative stress, inflammation, and M1 macrophage polarization. The reversal effect of ICAM1 overexpression on si‐USP7 confirmed that USP7 promoted LPS‐induced HPMEC injury by increasing ICAM1 expression. In addition, we demonstrated that USP7 had positively effect on NF‐κB pathway, which was consistent with the research of Zhao et al. [20].

Collectively, our study suggests a potential molecular mechanism that regulates pediatric ARDS progression. Our results pointed out that USP7 promoted ICAM1 protein expression through deubiquitination, which in turn accelerated LPS‐induced HPMEC injury. This finding provides a new molecular target for treating pediatric ARDS.

## Author Contributions

Jing Li performed experiments and wrote the manuscript. Jing Wu collected and analyzed the data. Lili Zhao conceived and designed research. Lian Liu contributed the methodology and edited the manuscript. All authors reviewed the manuscript.

## Conflicts of Interest

The authors declare no conflicts of interest.

## Data Availability

The data that support the findings of this study are available from the corresponding author upon reasonable request.
